# Introduction, adaptation and characterization of monk fruit (*Siraitia grosvenorii*): a non-caloric new natural sweetener

**DOI:** 10.1038/s41598-021-85689-2

**Published:** 2021-03-18

**Authors:** Shivani, Babit Kumar Thakur, C. P. Mallikarjun, Mitali Mahajan, Priya Kapoor, Jigyasa Malhotra, Rimpy Dhiman, Dinesh Kumar, Probir Kumar Pal, Sanjay Kumar

**Affiliations:** 1 Division of Agrotechnology, Council of Scientific and Industrial Research-Institute of Himalayan Bioresource Technology (CSIR-IHBT), Post Box No. 6, Palampur, HP 176 061 India; 2 Division of Chemical Technology, Council of Scientific and Industrial Research-Institute of Himalayan Bioresource Technology (CSIR-IHBT), Post Box No. 6, Palampur, HP 176 061 India; 3 Division of Biotechnology, Council of Scientific and Industrial Research-Institute of Himalayan Bioresource Technology (CSIR-IHBT), Post Box No. 6, Palampur, HP 176 061 India; 4 Academy of Scientific and Innovative Research (AcSIR), Ghaziabad, 201002 India

**Keywords:** Plant domestication, Plant physiology, Plant sciences

## Abstract

*Siraitia grosvenorii,* an herbaceous perennial plant, native to the southern parts of China, is commonly used as a low-calorie natural sweetener. It contains cucurbitane-type triterpene glycosides known as mogrosides. The extract from monk fruit is about 300 times sweeter than sucrose. In spite of its immense importance and International demand, *Siraitia grosvenorii* (Swingle) is not commercially cultivated outside China since scientific information for cultivation of this species is lacking. Planting material of monk fruit plant was not available in India. Thus, the seeds of monk fruit were introduced in India from China after following International norms. Then the experiments were conducted on different aspects such as seed germination, morphological and anatomical characterization, phenology, flowering and pollination behaviors, and dynamic of mogroside-V accumulation in fruit. The hydropriming at 40 °C for 24 h was found effective to reduce the germination time and to increase the germination rate (77.33%). The multicellular uniseriate trichomes were observed in both the leaf surfaces, however, higher trichomes density was observed in the ventral surface of males compared to females. The microscopic view revealed that the ovary was trilocular (ovary consists three chambers) having two ovules in each chamber or locule. Most of the fruits were globose or oblong type with 5–7 cm in length and 4–7 cm diameter. Mogroside-V content in fruit at 80 days after pollination was 0.69% on dry weight basis. The rate of increase of mogroside-V accumulation from 50 to 70 days was very slow, whereas a sharp increase was observed from 70 to 80 days. The higher receptivity of stigma was observed with fully open flowers. The floral diagram and formula have also been developed for both male and female flowers. Our results highlighted that monk fruit can be grown in Indian conditions.

## Introduction

*Siraitia grosvenorii* (Swingle) C. Jeffrey ex A. M. Lu & Zhi Y. Zhang commonly known as Monk fruit or Luo Han Guo, is an herbaceous perennial plant which belongs to the family of Cucurbitaceae. It is native to the southern parts of China prevalent in Yongfu, Longsheng and Lingui Counties in northern Guangxi province, and also distributed in Guizhou, Hunan, Guangdong and Jiangxi provinces^1–3^. These provinces have approximately 70% mountainous area with an annual mean temperature of about 16–20 °C and annual average precipitation is 1500–2002 mm^4^. The planting areas of monk fruit are generally distributed at an altitude range of 200–800 m with slopes gradient greater than 15°^4^. In China, it has been being cultivated in particular regions for more than 200 years^3^. The economic part of the species is fruit, which contains a sweet, fleshy, and edible pulp that is being widely used in traditional Chinese medicine for more than 300 years for the treatment of lung congestion, colds and sore throats^5,6^. It has been reported in Chinese Pharmacopoeia, since 1977. The monk fruit contains cucurbitane-type triterpene glycosides known as mogrosides. There are different types of mogrosides namely mogroside-III (MIII), mogroside-IVA (MIVA), mogroside-IV (MIV), mogroside-V (MV), iso-mogroside V (IMV), 11-oxomogroside-V (OMV) and siamenoside I (SI) etc. The extract from monk fruit have been estimated to be about 300 times as sweet as sucrose^7^, and is generally recognized as safe^8,9^.

Among the known mogrosides, mogroside-V is extremely sweet and low in calories^10,11^. Modern medicinal research shows that the ripe fruit extract and individual compound (especially mogroside-V) have anti-tumor^12,13^, antidiabetic^14–19^, anti-inflammatory^20^ and anti-oxidative^21^ properties. As the world working towards betterment of health and fitness, there is a huge need of natural, high potency and low or zero-calorie sweeteners over conventional dietary sweeteners in the food and beverage industries^22^. Due to intense sweetness, monk fruit becomes second important source of natural sweeteners right after *Stevia rebaudiana*^23^. Global monk fruit market forecasts that the period of 2019–2026 is expected to register a steady Compound Annual Growth Rate (CAGR) of 4.37%^24^. However, its market share is small as compared to the alternative sweetener market due to the limited supply, which remains about 2.2%^25^.

This species is not widely cultivated due to lack of agronomic practices, non-availability of quality planting material, poor adaptability, and less scientific knowledge, which are the main reasons for the limited supply. During the twentieth century, the 1^st^ attempt had been made for domestication in U.S. by Professor G.W. Groff^26^. However, the flowers did not appear may be due to the high summer temperature^1^. Till date, there is no evidence of the species being domesticated and cultivated in other countries than China^27,28^. The main reasons for its failure are inherent heterozygosity^29^ among the seed raised plants, dioecious in nature^30^, slow and low germination rate due to hard seed coat. Natural pollination is almost impossible due to the specific male and female flower structure and the sticky pollen grains, which cannot be pollinated by wind or insects^29^. Moreover, life of flower is short and intense, and pollen viability is affected over time^29^. Also, there are conventional techniques of asexual reproduction such as grafting and rooted cuttings, but they are time consuming, sensitive to viral infections and, often tedious and impractical for large scale use^31,32^. All these factors are the bottleneck for any crop for commercial cultivation. Simultaneously, the natural populations of monk fruit are rapidly declining due to over-exploration and habitat fragmentation^33^. Few studies have been conducted on chemistry^6,33–38^ and molecular aspect of monk fruit^39–41^. However, agronomic and physiological aspects are lacking. Thus, to meet the increasing demand in the international markets, there is an urgent need to domesticate and to develop Good Agricultural Practices for introduction in new areas. Simultaneously, it is necessary to understand the basic physiology, life cycle and growth behaviors for any species to introduce in new ecosystem. However, this type of information is lacking in the literature. Therefore, our main objectives were (1) to understand the complete life cycle for its adaptations in new environmental conditions; (2) morphological, anatomical, and chemical characterization to understand whether it is behaved like native places. Thus, a numbers of experiments were conducted under controlled and open field conditions.

## Result and discussion

### Seed germination studies

The data presented in Table 1 revealed that time required for germination and germination rate were significantly (*P* ≤ 0.05) affected by the pre-sowing seed stratification. Averaged across the duration, the days required for germination were reduced significantly (*P* ≤ 0.05) from 19 days with normal water to 12 days with hot water treatment. This result could be due to the fact that hot water increased seed coat permeability for water to increase seed hydration and for gaseous exchange. Irrespective of water temperature, hydropriming for 48 h significantly (*P* ≤ 0.05) reduced the germination time compared with 24 and 72 h. The interactions between hydropriming and duration of priming on germination time and germination rate were significant (Table 1). Seeds, hydroprimed at 40 °C or room temperature for 48 h, reduced the germination time by 20 days compared with hydropriming at room temperature for 24 h. However, when seeds were hydroprimed at 40 °C, there were no significant differences between hot water and normal water on the duration for germination time. Similarly, interaction effect between water temperature and duration of hydropriming on germination rate was found significant. Germination rate was significantly (*P* ≤ 0.05) increased from 56.89% with 24 h to 76.89% with hydropriming at 72 h under room temperature condition. However, when seeds hydroprimed at 40 °C, the germination rate was reduced at 49.78% with 72 h from 77.33% with 24 h priming. Thus, the best interaction result was obtained from hydropriming with hot water for 24 h (Table 1). The positive effects of seed priming have been noticed previously to increase the germination rate for many species^42,43^. In this experiment, low germination rate with long hydropriming (72 h) at 40 °C was probably due to the damage of cell. On the other hand, high germination rate with short hydropriming (24 h) at 40 °C was probably due to the fact that physiologically active state of pre-germinated seeds was improved. Priming with hot water is known to increase imbibition, which stimulates germination related biochemical activities, and ultimately weaken the endosperm^44,45^.

**Table 1:** Effect of seed stratification on date of sprouting and germination rate of monk fruit.

Hydropriming (H)	Duration (h) of hydropriming (D)	Germination time (day)	Germination rate (%)
Normal water	24	31.0	56.89
	48	11.0	60.44
	72	14.0	76.89
	Average	19.0	64.74
Hot water	24	12.0	77.33
	48	11.0	68.00
	72	13.0	49.78
	Average	12.0	65.04
Averages across water temperature	24	21.5	67.11
	48	11.0	64.22
	72	13.5	63.33
SEm ± for hydropriming (H)		0.63	1.30
CD (*P* = 0.05) for hydropriming (H)		1.96	NS
SEm ± for duration of hydropriming (D)		0.77	1.59
CD (*P* = 0.05) for duration of hydropriming (D)		2.40	NS
SEm ± for H × D		1.09	2.25
CD (*P* = 0.05) for H × D		3.39	7.02

### Morphological study of different parts of Monk fruit

#### Vegetative parts

**Figure 1:**
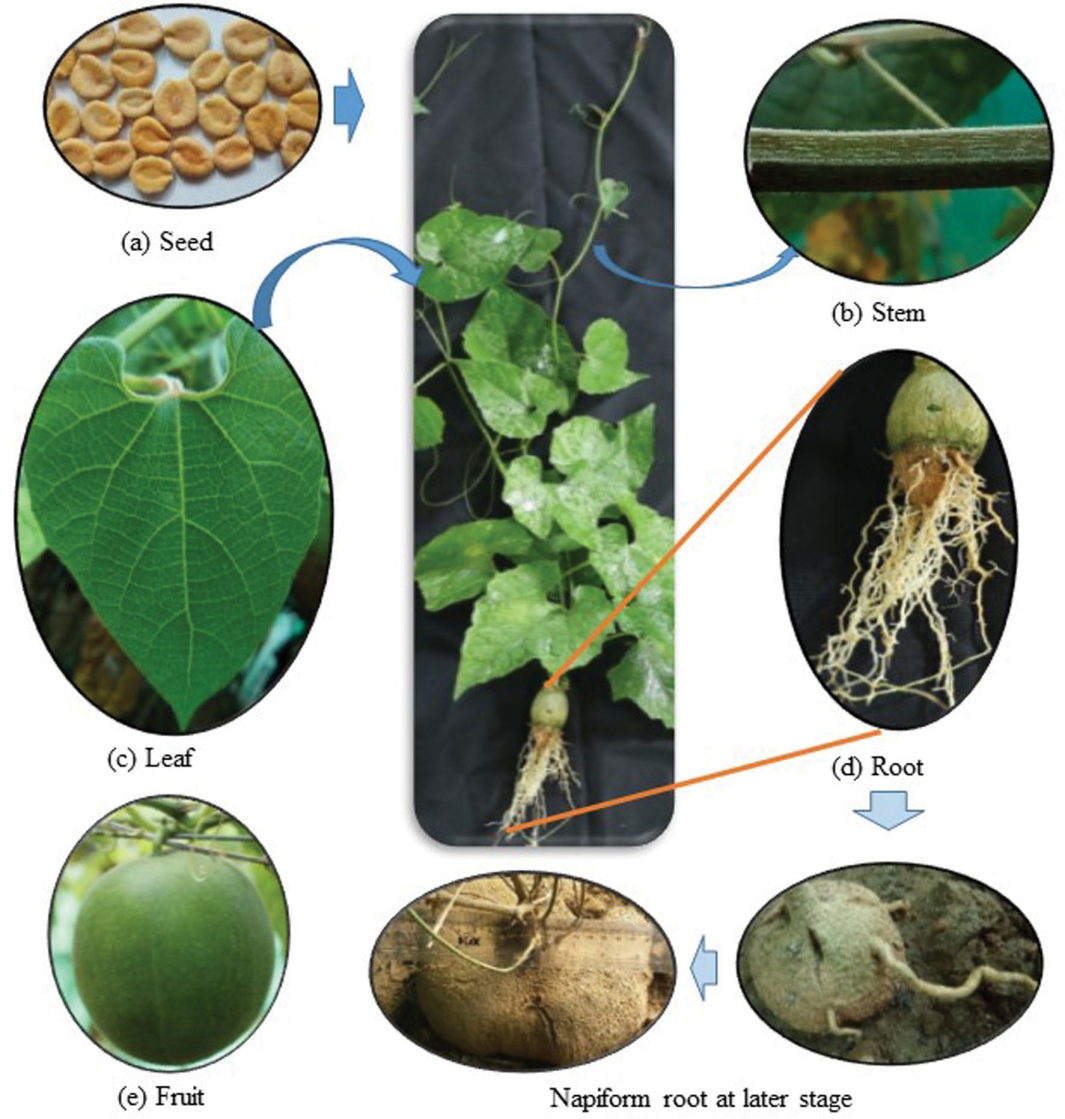
Different parts of *Siraitia grosvenorii*, seed **(a)**; pentangular stem **(b)**; heart shaped leaf **(c)**; napiform root **(d)**; and fruit **(e)**.

At both the vegetative and reproductive stages, morphological studies were done to understand the plant adaptations and behavioral changes in Palampur conditions. It has been observed that mature seeds of monk fruit show radial striae and furrows while side view possess erose margins (Fig. 1a), which are similar to earlier description given by Swingle^1^. Epigeal or epigeous germination (*Epi* = upon; *geal* = soil) behavior was observed, which is the established rule in Cucurbitaceae family^46,47^. In Palampur conditions, the length of plant was recorded up to 15 m, whereas 3–5 m has been reported from China^1,48^. Its stem is weak, herbaceous, climber, angular, and green in color (Fig. 1b). Stem is pentangular with ridges and furrows. Stem surface is covered by small hair like outgrowth. It has alternate type of branching pattern. The length of internodes was up to 30 cm, whereas only 3–9 cm long internodes have been reported in Chinese conditions^1^. A few members of Cucurbitaceae are perennial in nature, out of which 50% are dioecious^49,50^. Monk fruit is one of the perennial dioecious of Cucurbitaceae.

Though, the leaf’s shape was not different from earlier reports^1,48^, the size was relatively larger under Palampur conditions (Fig. 1c). We have registered leaf size up to 32 cm long and 25 cm wide. However, earlier it has been reported about 12–23 × 5–17 cm^1,48^. At initial growth stage, branched tap root was noticed, but at active growth stage it was looked like a napiform root, which was about 10–20 cm in diameter (Fig. 1d). In contrary, fusiform and sub-globose types of roots (10–15 cm) have been reported in earlier reports^1,48^. The matured fruits were pepo type, which is an indehiscent fleshy single-celled many-seeded berry (Fig. 1e). Most of the fruits were globose or oblong with 5–7 cm in length and 4–˗7 cm diameter in Palampur conditions, which are little bit smaller than earlier reported (6-11 cm long and 3–-8 cm broad) from China ^1,48^. The variation in fruit shape among the cultivars and/or populations has been already reported. The square-round shape has been reported for Pufengqingpi cultivar^51^.

#### Reproductive parts

**Figure 2:**
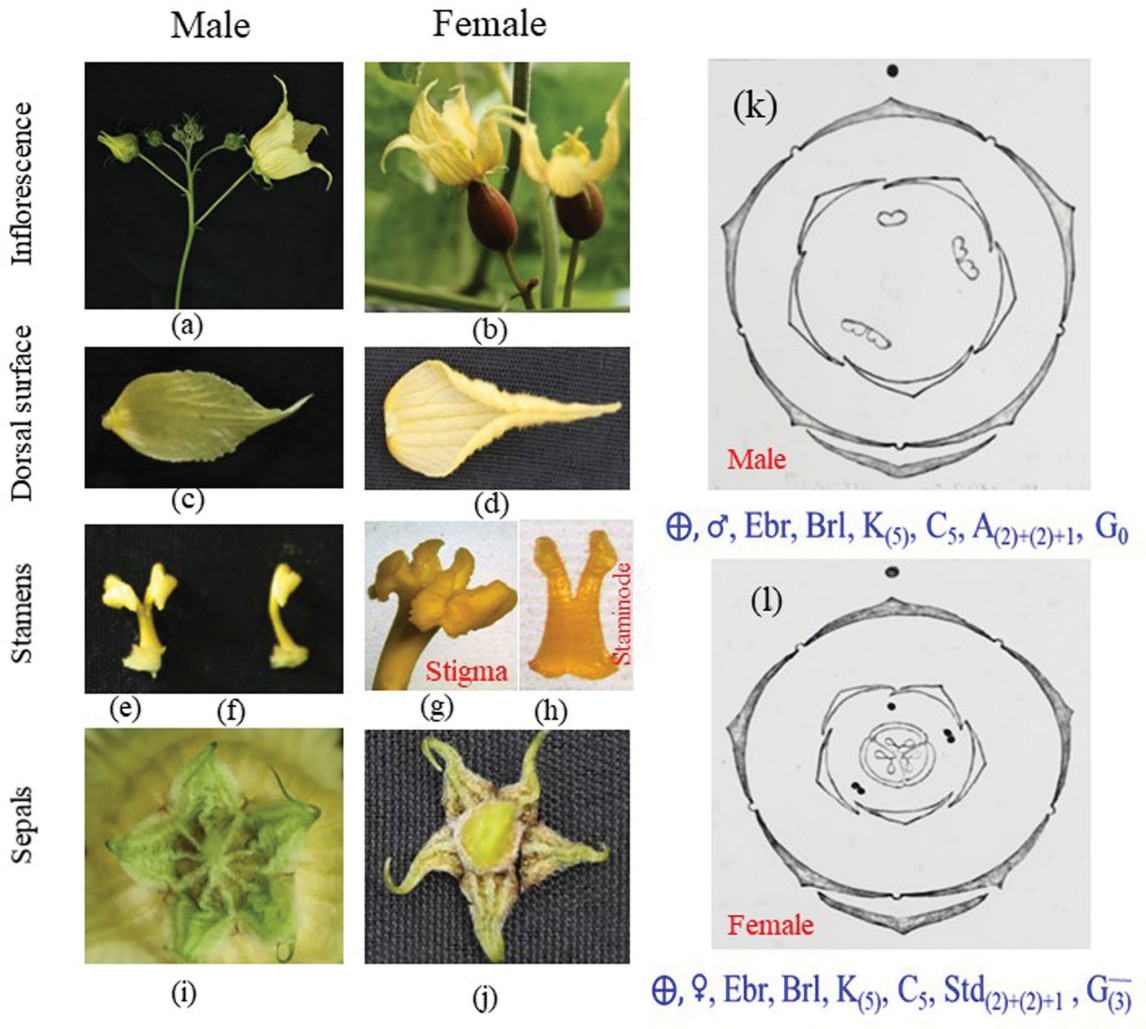
Different parts of *Siraitia grosvenorii* flowers, male inflorescence **(a)**; female flower **(b)**; male petal dorsal surface **(c)**; female petal dorsal surface **(d)**; stamen compound **(e)**; simple stamen **(f)**; stigma **(g)**; compound staminode **(h)**; male sepals **(i)**; and female sepals **(j)**. Floral formula of male **(k)** and female **(l)** flower.

The unisexual flowers were observed in the axil of leaf. Male flowers were in racemose inflorescence. Female flowers were arranged either solitary in the axil of leaf or in racemose cluster (2–5 in number). Both flowers were characterized by yellow in color, pedicellate, ebracteate, incomplete, actinomorphic, pentamerous, and regular (Fig. 2a,b). Bract was not observed. Both male and female flowers were incomplete since androecium and gynoecium were not found in the same flower. Female flowers were epigynous with floral parts such as the petals and stamens were attached to the upper part of the ovary. Syncarpous ovary with fused carpels was observed, and inferior ovary positioned below the floral parts. In female flower, two groups of two fused staminodes (Fig. 2h) and one single staminode were observed. In our studies, simple style and trifid stigma with bifid lobes were observed (Fig. 2g), whereas 3-lobed and sometimes each bifid lobe have been reported from China^1^. At the time of full blooming stage, the ovary size was recorded about 15 × 8 mm. Five sepals which were observed in both the flowers (male and female), were green in color and united at the base (Fig. 2i,j). Five free-petals were arranged in quincuncial aestivation, in which two petals or sepals were positioned internally and two petals or sepals were placed externally, and the fifth part was situated externally at the margin and two of the petals (nos. 1 and 2). In spite of five stamens in male, they appeared as three with two groups of two fused stamens forming two compound stamens (Fig. 2e) and one simple stamen (Fig. 2f). In male flower, anther possesses a single pollen-locule of S-shaped with outer descending arm (longer) and inner ascending arm (shorter). Pistillode (sterile pistil) is not found in male flower, although it is usually present in many genera of Cucurbitaceae. Corolla size of male flower was up to 4.5 cm long and 3.8 cm wide (Fig. 2c), which was larger than earlier reported by Anmin and Jeffrey^48^. Based on our studies, the floral diagram and formula (Fig. 2) have been developed for both male and female flowers.

### Anatomical study of different parts

#### Vegetative parts

**Figure 3:**
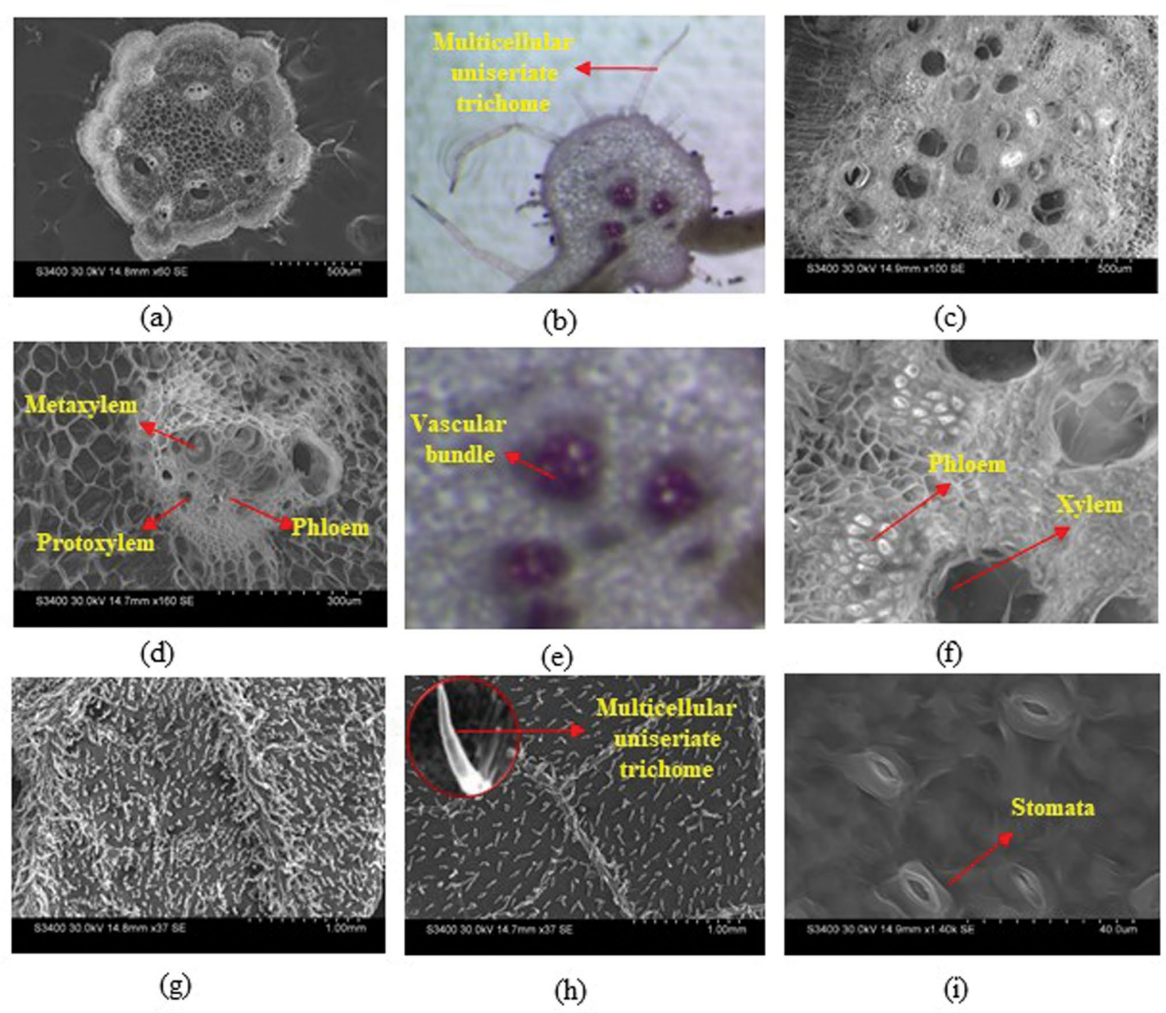
Scanning electron microscopic (SEM) micrographs of transverse sections of stem **(a)**; leaf through midrib **(b)**; root **(c)**; vascular bundle of stem showing metaxylem, protoxylem and phloem **(d)**; vascular bundle of leaf **(e)**; root section showing xylem and phloem **(f)**; lower surface of male leaf **(g)**; lower surface of female leaf with magnifying view of trichome **(h)**; and stomata on lower epidermis **(i)**.

The structural organizations of leaf, stem and root of monk fruit were studied (Fig. 3). It was observed that stem was pentangular with ridges and furrows, and vascular bundles were arranged in two rings at the base of each furrow and ridge (Fig. 3a). Vascular bundles were bicollateral, closed, and endarch (metaxylem present towards the periphery and protoxylem towards the center). Scanning electron microscopy (SEM) images revealed that protoxylem contained narrow vessels and cell wall thickenings in the form of rings or helices, whereas metaxylem contained larger vessels and cell wall thickenings in the form of transverse bars like ladder (Fig. 3d). Transverse section of leaf through midrib region (Fig. 3b) showed hydrocentric or concentric amphicribral vascular bundles (xylem lies towards the center and are completely surrounded by phloem) (Fig. 3e). Both the leaf surfaces were covered with hair like outgrowths known as trichomes, which are multicellular uniseriate (Fig. 3g,h). Paracytic type stomata were observed on the ventral surface (lower epidermis) of leaf. The guard cells were surrounded by two subsidiary cells that positioned parallel to the long axis of pore and guard cells (Fig. 3i). Differences in trichomes density were observed between male and female plant leaves (Fig. 3g,h). The higher trichomes density was observed in ventral surface of male leaves compared with ventral surface of female leaves (Fig. 3g,h). However, higher stomatal density was observed in ventral surface of female leaves than male leaves. Transverse section of lateral root showed that the vascular bundles were radial (Fig. 3c). The closed view of xylem and phloem (Fig. 3f) showed that xylem was in ring shaped and phloem was small compressed cells.

#### Reproductive organs

**Figure 4:**
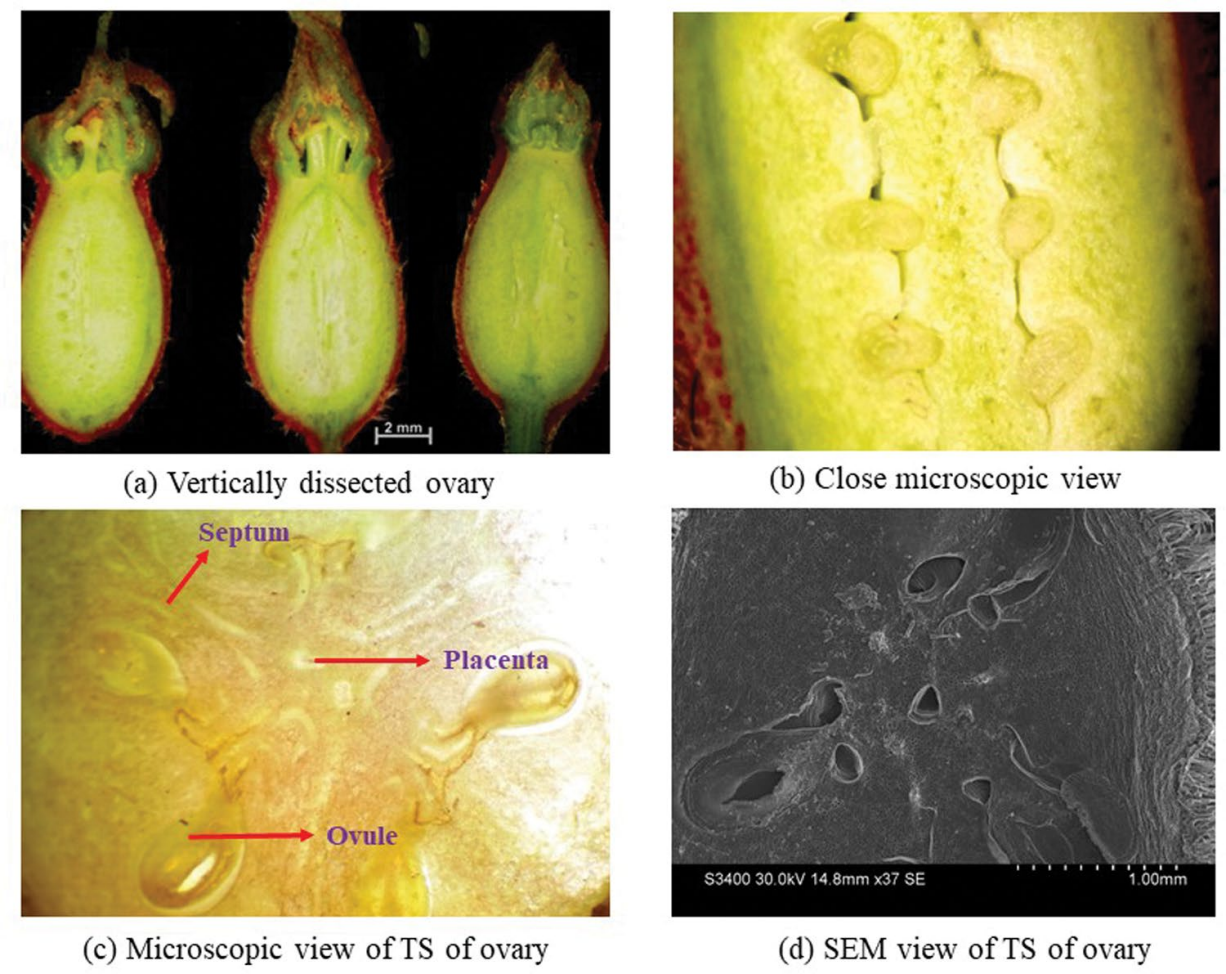
Vertically dissected view of monk fruit ovary **(a)**; closed view of dissected ovary showing the arrangement of ovules **(b)**; microscopic view of T.S. of ovary showing axile placentation (40x magnification) **(c)** and SEM view of T.S. of ovary **(d)**.

Male and female flower petals surfaces of monk fruit were covered with the small multicellular uniseriate hair like trichomes. Close microscopic view of vertically dissected ovary is presented in Fig. 4a,b. Microscopic view reveals that ovary is trilocular (ovary consists three chambers) having two ovules in each chamber or locule (Fig. 4c,d). Similar observations have been reported by Swingle^1^ through serial microtome cross-sections study of ovary. Ovules were borne at the center of an ovary on an axis formed from joined septa showing axile placentation (Fig. 4a,b).

### Reproductive stage and behaviors

We have categorized the reproductive stage in five distinct phases such as (i) bud stage (ii) partially open (iii) fully open flower (iv) wilting stage and finally (v) senescent stage (Table 2). Our study revealed that ovary at bud stage of female flower was reddish green in color, size varied from 1–5 mm in length and stigma was not visible. In partially opened flower, the color of the ovary was reddish green, 7–9 mm in size, and stigma lobes were visible and yellow in color. In fully open flower, ovary remained reddish green; 10–15 mm in size, pedicel 4–9 mm and stigma was fully visible and yellowish in color. In wilted stage, color of the ovary was reddish brown, 7–9 mm in size, pedicel 4–7 mm, stigma was fully visible and yellow in color. At senescent stage, ovary converted into brown in color, 4–6 mm in size, pedicel also 4–6 mm, and stigma was fully visible with blackish yellow in color (Table 2).

**Table 2:** Developmental stages of male and female flowers of *Siraitia grosvenorii* based on major morphological characteristics.

Developmental stage	Plant type	Physical appearance	Bud			Flower pedicel (length in mm)	Stigma		Anther		Pollen condition
			Petal condition	Color	Size (mm)		Visibility	Color	Color	Texture	
Stage I	Male	Small green bud	Close	Reddish green	1–4	1–5	–	–	Bright yellow	Smooth	Not available
	Female	Small green bud	Close	Reddish green	1–5	1–2	Not visible	–	**–**	**–**	**–**
Stage II	Male	Partially open flower	Opening	Reddish green	3–6	3–15	–	–	Bright yellow	Light dusty	Some adhere to petals
	Female	Partially open flower	Opening	Reddish green	7–9	1–5	Stigma lobes can visible	Yellow	**–**	**–**	**–**
Stage III	Male	Fully open flower	Open	Reddish green	9–16	10–15	–	–	Bright yellow	Dusty	Available and extensively attached
	Female	Fully open flower	Open	Reddish green	10–15	4–9	Fully visible	Yellow	**–**	**–**	**–**
Stage IV	Male	Wilted flower	Closing	Reddish green to brown	9–12	10–15	–	–	Light yellow	Little dusty	Few pollen are left
	Female	Wilted flower	Closing	Reddish brown	7–9	4–7	Fully visible	Yellow	**–**	**–**	**–**
Stage V	Male	Senescent flower	Close	Reddish green to brown	9–10	10–15	–	–	Light yellow	Smooth	Very few pollen are left
	Female	Senescent flower	Close	Brown	4–6	4–6	Fully visible	Blackish yellow	**–**	**–**	**–**

**Figure 5:**
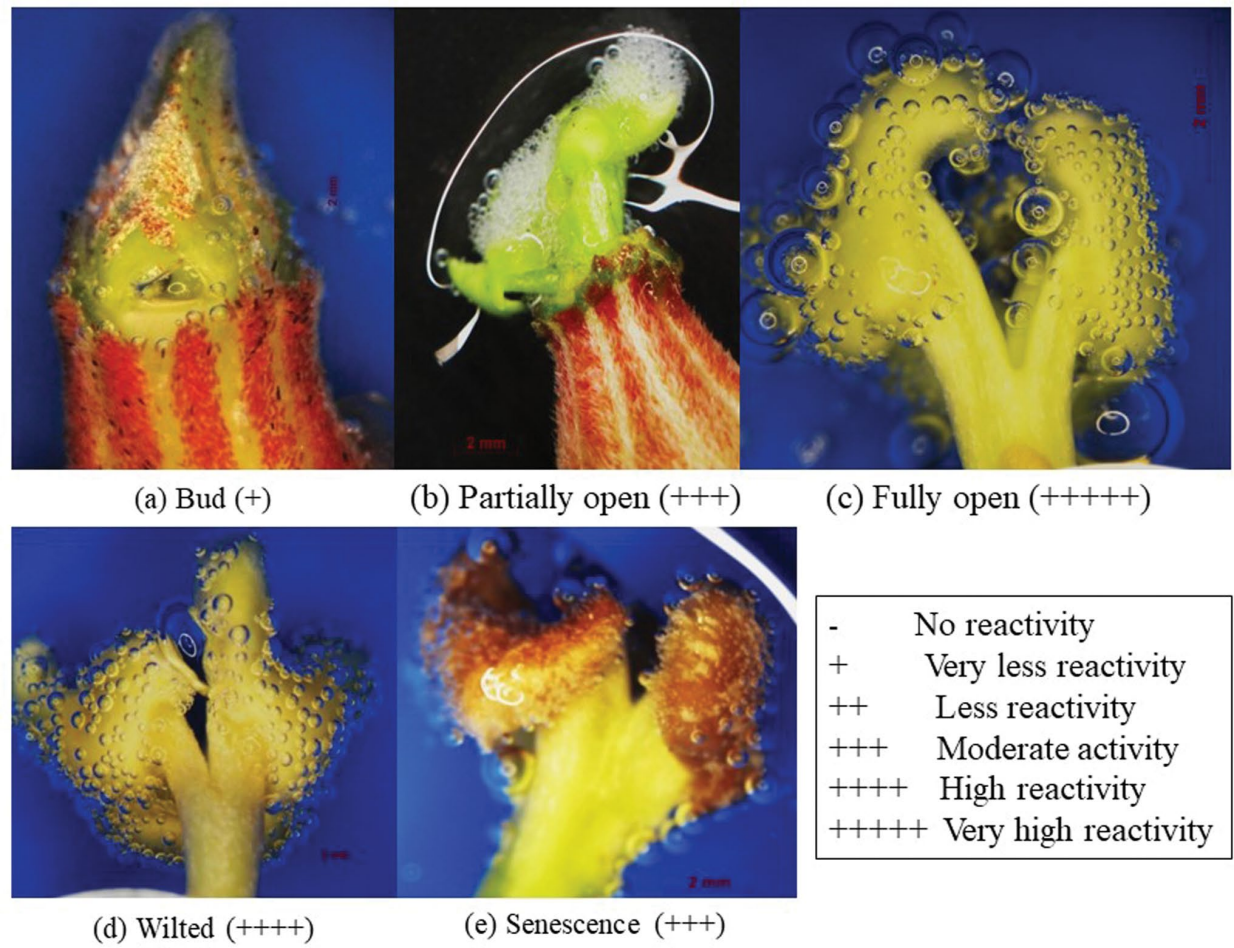
Microscopic view of stigma receptivity at different stages, bud **(a)**; partially opened flower **(b)**; fully opened flower **(c)**; wilted flower **(d)**; and senescent flower **(e)**.

Receptivity of stigma was also studied and presented in Fig. 5. The bubbling from stigma showed the receptivity of stigma (Fig. 5). As per our observation, anthesis stage (full opened) of the female flower bud was found the best stage for the receptivity of pollen grains for fertilization (Fig. 5c). Thus, it is clear that fully open flower stigma has higher receptivity than others, and it is the best stage for fertilization for fruits setting. It could be an immense practical important for manual pollination during commercial cultivation as it is a dioecious plant. In male flower, size, color, length of the pedicel, anther texture, color and availability of pollen grains at different stages have also been observed (Table 2). During male bud development, it was noticed that color of the bud was changed from reddish green to brown during different stages. The length of petiole was also varied, and it was up to 15 mm. Color of the anthers was bright yellow and changed to light yellow at wilted and senescence stage. Pollens were available in fully open male flower, yellow in color, bright and sticky, after that they became black in color at wilted and senescent stage. Thus, it is clear that the stage III (fully open male flower) is best stage for highly viable pollens as per our observation.

### Flowering, pollination behavior and fruit development

**Figure 6:**
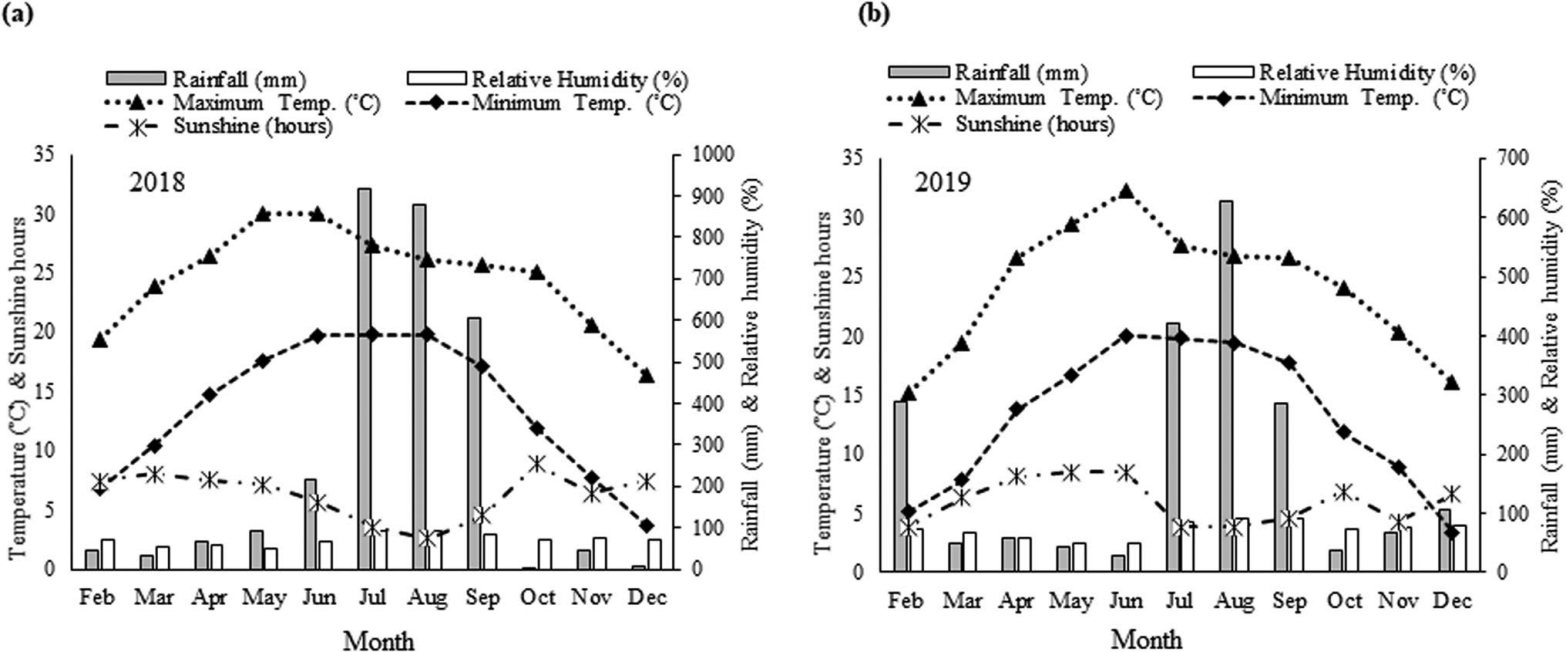
Monthly mean maximum and minimum temperatures (°C), relative humidity (RH%) rainfall (mm), and sunshine hours (SS) during the plantation and study seasons of 2018 **(a)** and 2019 **(b)**.

The flowering pattern, pollination behavior and fruit setting time were also recorded to draw a complete life-cycle of monk fruit in a new agro-climatic conditions. The data showed that male flowers were appeared earlier than female flowers during the experimental years. This result was probably due to the fact that male plant entered into the reproductive phase earlier compared with female plant. In another study, it has been reported that male flowers bloom 2 weeks before the first female flowers in *Momordica charantia* during long day conditions^52^. The shifting of flowering season was also observed in this investigation. In 2018, flowering season started in the mid of July, and September was peak month; however, in 2019, flowering season started in mid of May, and August was peak month. The shifting of flowering season and differences in duration of flowering might be due to age difference of the plant or changes of weather parameters (Fig. 6), which influenced the flowering related physiological activities. In contrast, flowering season of monk fruit is May–July in China^48^. This difference might be due to climatic factors.

**Figure 7:**
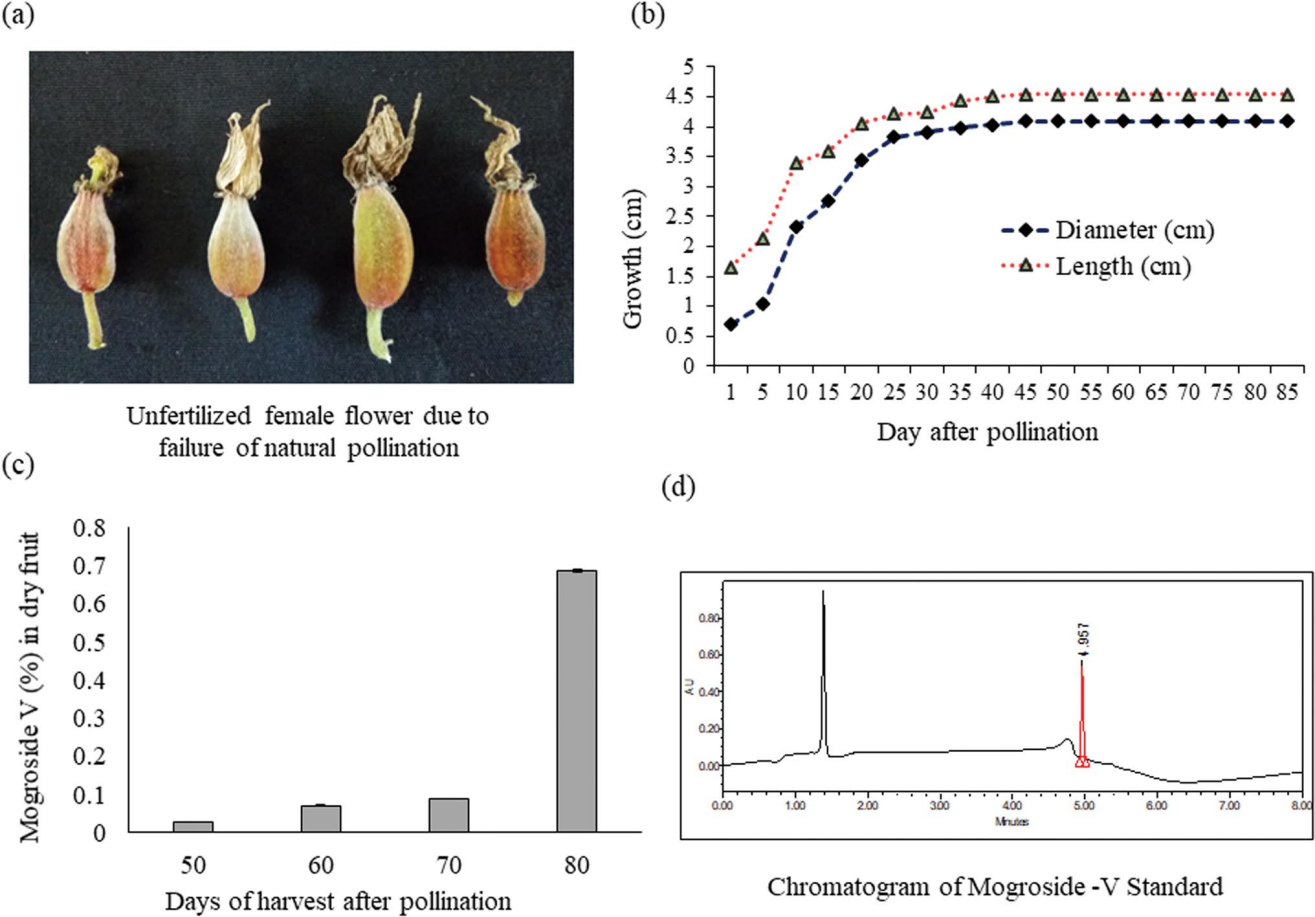
Unfertilized female flower due to failure of natural pollination **(a)**; growth pattern of monk fruit **(b)**; dynamic of mogroside-V accumulation in fruits **(c)**; and chromatogram of mogroside-V standard **(d)**.

To test the possibility of natural pollination, few numbers of female flowers were left without disturbance and any physical obstacle in the field conditions. All the female flowers were dried up along with the petals (Fig. 7a). Vectors like honeybees and ants were seen in male flowers, but these insects did not visit female flowers. This phenomenon indicates that the chance of absence of nectar in female flower of monk fruit is very high. Similar observation has been reported in *Momordica species,* in which male flowers possess nectar, pollen and fatty oils, while female flowers contain only fatty oil^53^. The another possible cause of failure for natural pollination was short life of female flowers (2 days). Thus, manual pollination technique is practiced in China for fruit setting^54^. The manual pollination technique was also adopted in our studies. After fertilization, ovary was started to develop into fruit, and the length and diameter of ovaries were increased steadily at initial stage; however, no changes in size were observed after 40 days of pollination (Fig. 7b).

### Chemical characterization and dynamics of mogroside-V accumulation

The understanding about the dynamics of mogroside-V accumulation in fruits in a particular location is required to harvest quality fruits. The data revealed that the accumulation of mogroside-V was significantly (*P* ≤ 0.01) affected by the stage of fruit harvesting (Fig. 7c). The representative chromatogram for the mogroside-V standard has also presented in Fig. 7d. The maximum mogroside-V content (0.69% in dry sample) was recorded with fruit harvested at 80 days after pollination, which was significantly (*P* ≤ 0.01) different from fruits harvested at 50, 60, and 70 days. The magnitude of mogroside-V accumulation in fruits was 80 > 70 > 60 > 50 days after pollination in this present experiment. The rate of mogroside increase from 50 to 70 days was very slow, whereas sharp increase was observed from 70 to 80 days (Fig. 7c). In earlier study, it has been reported that the range of mogroside-V content in dried fruit is from 0.5–1.4%^6^. This difference was probably due to harvesting time, seasonal variation and habitat^35^. Tang et al.^33^ also reported that the mogroside-V content was sharply increased from 50 to 70 days after fertilization, and levels were constant after 85 days. At the early stage of fruit development, the mogroside II E and mogroside III are developed, which are bitter and tasteless, respectively, and these gradually decrease with maturity of fruits^35^. Therefore, the fruit should not be harvested based on size since the distinction of harvesting stages by shape is difficult. It is clear from the present study that the fruit harvesting time is one of the key agronomic factors to maintain the quality of fruits.

## Conclusions

It is fact that monk fruit has been successfully grown and harvested quality fruits in Indian soil. The hydropriming at 40 °C for 24 h is the effective technique to reduce the germination time and to increase the germination rate. Based on the physical appearance the reproductive stages have categorized in five distinct phases such as (i) bud stage (ii) partially open (iii) fully open flower (iv) wilting stage and finally (v) senescent stage. It is also validated that possibility of natural pollination is very less or nil. Thus, for commercial cultivation, manual pollination technique should be adopted at fully open flowering stage as the stigma receptivity is higher at this stage. This study also examined that the quality of fruits is largely governed by the harvesting time. The fruit should not be harvested before 80 days after pollination to maintain the quality in terms of mogroside-V. Further studies are required to understand deep physiology and different aspect of agronomy particularly role of nutrient management.

## Materials and methods

### Introduction of planting material and experimental location

Planting material of monk fruit plant is not available in India for the cultivation. Thus, the seeds of monk fruit were introduced in India from China, through Indian Council of Agricultural Research-National Bureau of Plant Genetic Resources (ICAR-NBPGR), New Delhi, with Import Permit No. 168/2017, in 2018. Seeds were received from ICAR-NBPGR after quarantine clearance. As per guideline, the plants were initially grown under controlled conditions at CSIR-IHBT, Palampur, India for one year with proper vigilance of ICAR-NBPGR. During this period no pathogen or disease has been noticed. After that, the plants were grown in poly-house as well as open field conditions at experimental field of CSIR-IHBT, Palampur (32° 06′ 05″ N; 76° 34′ 10″ E) at an altitude of 1393 amsl. Soil of the experimental field is slightly acidic in nature. The weather parameters such as rainfall, maximum and minimum temperatures, relative humidity, and sunshine hours of the plantation site during the growing seasons are presented in Fig. 6.

### Seed germination studies

For seed germination study, 450 seeds were randomly selected from the population of 1 kg seeds. Selected seeds were washed with tap water to remove all the impurities attached with them. Experiment was conducted with six treatment combinations comprising stratification of seed with hot (40 ± 2 °C) and normal water (~ 20 ± 2 °C) at different durations (24, 48 and 72 h). For each treatment, 25 seeds were used with three replications. The seeds were kept for germination in petri dishes over moist filter paper. All dishes were kept in the seed germinator with temperature and relative humidity ranged from 25 to 27 °C and 85–90%, respectively. The filter papers of petri dishes were regularly moistened with distilled water and monitored daily for radicle emergence. The numbers of germinated seeds for each treatment were counted every day up to 45 days.

### Morphological studies

For morphological analyses, 5 males and 5 female plants of monk fruit were randomly selected and tagged, which were free from all biotic and abiotic stresses. Different parts of plants such as leaf, stem, roots and flowers were randomly collected from the selected plants. Collected parts were washed with tap water to remove the impurities from their surfaces, and then keenly observed using light microscope (40× magnification) and scanning electron microscope (SEM). For microscopic studies, different parts of the monk fruits were fixed in FAA solution (formalin, acetic acid, 95% ethanol, distilled water, 10:5:50:35, v/v) under smooth vacuum for 1 h, then dried for 12 h at room temperature^55^. Ovary of the female flower was also dissected transversely, and observed under the microscope (40× magnification). Data were recorded for both male and female plants of monk fruit.

### Anatomical studies

For anatomical studies, different parts of male and female plants were collected from tagged plants. Samples were collected during both the vegetative stage and reproductive stage. The samples of leaves, stem and flowers were washed to remove the impurities from their surface with tap water. Anatomical studies of plants were done by using scanning electron microscope (SEM) to understand the difference between male and female plants. For microscopy study, fresh samples of monk fruit plant such as leaf, stem and root were collected. These different parts were dissected and mounted on the metal stub using sticky carbon disc and then coated with gold, finally samples were observed in SEM.

### Phenological and reproductive studies

The size and color of the ovary, length of the pedicel, stigma visibility and color of the female flower have been recorded at different stages. The bud size and color, flower pedicel, anther color and texture, and availability of pollens of the male flower have been recorded at different stages of development.

#### Stigma receptivity and pollination behavior studies

To study the stigma receptivity, female flowers were randomly selected and tagged with date of pollination. Tagged flowers were collected at five different stages based on physical appearance like bud stage, partially open, fully open, wilted and senescent stage of flower. Three tagged flowers were used at each stage. All the floral parts were removed and the stigmas from the flowers were kept on slide. A solution of 6% hydrogen peroxide was dropped on stigma and observed under microscope (40× magnification). The release of oxygen bubbles due to the direct action of oxygenated water with enzymes present in the stigma was considered as mark of receptivity of stigma^56^.

For pollination behavior studies, total twenty female buds of same age group were selected. Half of them were kept for natural pollination, and remaining half were manually pollinated. After opening of the female buds, data on changes of bud in size have been recorded time to time.

### Floral diagram and formula

To draw floral diagram of male and female flower, we observed the floral buds that were partially opened. Then, we pluck the floral buds after observing their anterior and posterior sides from the mother axis. In bracteolate flowers, bracteoles were drawn in section on the left and right sides of the diagram. The number of sepals and their arrangement in relation to the mother axis and aestivation were noted. Keeping all these points in view, transverse sections of sepals between the mother axis and the bracteole have been drawn. If sepals are odd in number, then sepal would be drawn either posterior or anterior to the flower, i.e., opposite the mother axis or opposite the bract, respectively. The different floral whorls of the flowers were represented in concentric circles, the sepals on the outermost circle, then, the petals, stamens and carpels towards the inner side. After drawing floral diagram, floral formula has been developed. Floral formula provides information regarding symmetry, sexuality and interrelationship of various floral parts viz., calyx, corolla, androecium and gynoecium.

### Chemical characterization and dynamics of mogroside-V accumulation

To know the mogroside-V content and its dynamics in fruits in Indian conditions the female flowers were tagged with date of pollination to harvest at a particular day. The fruits were harvested at 50th, 60th, 70th, and 80th day after pollination (DAP) for chemical analysis. For chemical analysis 100 mg of each powdered sample was taken and extracted in 70% of methanol by sonication method. Samples were sonicated for 10 min followed by centrifugation at 8000 rpm for 10 min. This process was repeated three times up to 5 mL of solvent. The samples were filtered through 0.22 µm syringe filter and transferred into vials for further analysis. Standards were prepared with 1 mg of mogroside-V obtained from Sigma Aldrich and dipped in 70% methanol, filtered with syringe of 0.22 µm and transferred into vials. The standards were run before the sample to observe the standard peaks of mogroside-V. Then, UPLC-PDA quantification of morgoside-V in samples was performed by Waters Acquity UPLC, H-class system. The analytical column used was Zorbax Eclipse Plus C18 column (2.1 × 150 mm, 1.8 µm). Detection wavelength was set at 205 nm. Elution was performed at a solvent flow rate of 0.25 mL/min. The gradient elution system was used, mobile phase A contained 0.1% formic acid in water, mobile phase B was acetonitrile (ACN). The gradient started from 0 min at 20% B; then from 0 to 1.50 min, 20% B; 1–3 min, linear gradient from 20 to 40% B; 3–3.50 min, 50% B; 3.50–6.50 min, 25% B, and 6.50–7.0 min, 20% B, then again mobile phase ran on initial conditions, 7–8 min, 20% B, to equilibrate column on initial conditions.

### Statistical analysis

The data on seed germination were analyzed statistically by analysis of variance (ANOVA) using Statistica 7 software (Stat Soft Inc., Tulsa, Oklahoma, USA). Means were separated by the least significant difference (LSD) value when the F-test was significant (*P* ≤ 0.05). In case of Mogroside-V, the LSD was considered to compare between means at *P* ≤ 0.01.
